# HTKS measure of executive function and self-regulation: two decades of progress in early childhood measurement

**DOI:** 10.3389/fpsyg.2026.1801156

**Published:** 2026-07-13

**Authors:** Megan M. McClelland, Sabrina A. Kenny, Samantha Didrichsen, Paige Noelle Herrera, Simone E. Halliday, Ahmad Ahmadi, Claire E. Cameron

**Affiliations:** 1 Human Development and Family Sciences, Hallie E. Ford Center for Healthy Children & Families, Oregon State University, Corvallis, OR, United States; 2 Hallie E. Ford Center for Healthy Children & Families, Oregon State University, Corvallis, OR, United States; 3 Department of Learning & Instruction, University at Buffalo, Buffalo, NY, United States; 4 Morgridge College of Education, University of Denver, Denver, CO, United States

**Keywords:** academic achievement, executive function, measurement, school readiness, self-regulation

## Abstract

This review article examines the evolution and impact of a widely used measure of early self-regulation and executive function (EF) in children: the Head-Toes-Knees-Shoulders (HTKS) task. We discuss the importance of SR and EF for children’s school success and provide an overview of how these constructs have been conceptualized and operationalized. After situating the HTKS among other SR/EF measures, we trace its development from the original Head-Toes Task, to the HTKS and the HTKS-R and, most recently, the HTKS-Kids digital app and describe how the HTKS has been applied across populations and contexts. We synthesize evidence highlighting its measurement properties (e.g., reliability, validity, developmental change, and associations with academic performance) across diverse cultural and developmental contexts. Special attention is given to the need to extend and validate the HTKS across various educational and practice settings, with an emphasis on the roles of educators in supporting equitable assessment practices. We discuss recent advancements in measurement, including gamified digital administration, adaptations for neurodiverse populations and extensions of the HTKS into adolescence and adulthood. Finally, we identify limitations and directions for research and practice.

## Introduction

Self-regulation and executive function (EF) have been among the most studied constructs in child development and developmental psychology over the past several decades (McClelland et al., 2015). Self-regulation refers to children’s capacity to manage attention, behavior, and emotions in support of goal-directed activity and plays a central role in school readiness and learning across academic and social domains (Blair and Raver, 2015; Edossa et al., 2018; Hernández et al., 2018; McClelland et al., 2018; McClelland et al., 2021; Moffitt et al., 2011; Robson et al., 2020). In early childhood, advances in a child’s overall self-regulation is often understood as arising from their maturing interrelated EF processes, particularly *inhibitory control* or the ability to suppress a dominant response in favor of a more adaptive one, *cognitive flexibility* or the ability to shift attention and strategies as demands change, and *working memory* or the ability to hold and manipulate information (Best and Miller, 2010; Blair, 2016; Nigg, 2017).

Recent meta-analytic work underscores some of the long-term benefits of well-developed self-regulation. Robson et al. (2020), synthesizing findings from 150 studies, concluded that self-regulation in preschool predicts academic performance and classroom engagement in the early grades, and academic functioning well into adolescence. Their findings support earlier longitudinal studies demonstrating that self-regulation uniquely contributes to children’s learning outcomes, even when accounting for IQ, background characteristics, and prior achievement (Duncan et al., 2007; Edossa et al., 2018; Fuhs et al., 2014; Schmitt et al., 2017; von Suchodoletz et al., 2013).

The broader child development literature highlights the malleability of self-regulation, suggesting that it is significantly shaped by environmental experiences (McClelland et al., 2015). On the one hand, children’s self-regulatory capacities may be constrained by factors such as genetic predispositions or exposure to adverse life circumstances; on the other, substantial evidence indicates that these skills can improve when children are provided with supportive environments and opportunities to practice regulatory behaviors. Such improvements have been linked to enhanced long-term well-being, including more optimal school and life outcomes (Blair and Raver, 2015; Howard and Williams, 2018; McClelland et al., 2015). Even modest gains in regulatory abilities during childhood have been associated with benefits extending into adulthood (Moffitt et al., 2011). This finding is inspiring, as chronic stress and adversities tax and constrain the neural development needed for strong self-regulation (Blair and Raver, 2015). At the same time, self-regulation may offer protective benefits for children facing such adversities. For example, among children from socioeconomically disadvantaged backgrounds, strong self-regulation predicts better academic outcomes in preschool and remains consistently beneficial (McClelland and Wanless, 2012).

Recognition of the malleability of children’s self-regulation skills has motivated a substantial body of research examining the effectiveness of self-regulation–focused interventions. Findings from this work have been promising across diverse samples of children, including children with disabilities and children from families facing socioeconomic hardships (Carr et al., 2014; Flook et al., 2015; Hahn-Markowitz et al., 2020; McClelland et al., 2019; Pandey et al., 2018; Schmitt et al., 2015; Welsh et al., 2020). Research has been strongest for near-transfer effects (e.g., Kassai et al., 2019) with some evidence for far-transfer effects (e.g., McClelland et al., 2019). Assessment of these skills is essential for the early identification of children in need of support, for informing the design and implementation of interventions, and for evaluating the effectiveness of intervention programs across diverse populations (McCoy, 2019).

Collectively, this evidence highlights the need for developmentally appropriate, scalable, and psychometrically sound tools capable of capturing young children’s self-regulation across diverse cultural, linguistic, socioeconomic, and neurodevelopmental contexts. Such tools must reflect the ways regulatory skills are expressed in everyday classroom activities while remaining sensitive to developmental variability. It is within this context that the *Head-Toes-Knees-Shoulders (HTKS)* tasks emerged and evolved over the past 20 years. The HTKS is designed to assess self-regulatory skills through a game-like format and has undergone multiple iterations to expand its age range, improve measurement precision, and enhance ecological validity for use with diverse populations. As a result, the HTKS and its revised versions (e.g., HTKS-R) are now widely used behavioral assessments of self-regulation around the world and available in over 31 languages (Kenny et al., 2023; Korucu et al., 2025).

The present manuscript traces the development and progression of the HTKS, from the original Head-Toes Task (Cameron Ponitz et al., 2008; McClelland et al., 2007a), to the more complex structure of the HTKS (Cameron Ponitz et al., 2009; McClelland et al., 2014), to the downward extension offered by the HTKS-R (Gonzales et al., 2021; McClelland et al., 2021), and most recently to emerging adaptations designed to extend its relevance and utility across populations and contexts, including digital delivery (HTKS-Kids; Cameron et al., 2024), adaptations for adolescents (Fernandes et al., 2023), adults (Cerino et al., 2018), and neurodiverse learners (Cameron et al., 2025). Our goal is to illustrate how the iterative development of the HTKS responds to longstanding measurement challenges related to developmental variability, ecological validity, contextual sensitivity, and equity. This synthesis offers researchers who currently use the HTKS, and others seeking developmentally appropriate, scalable measures of self-regulation, a clear account of the task’s theoretical foundations, psychometric evolution, and appropriate applications, thereby supporting more informed measure selection, interpretation, and future development in self-regulation research. It is important to note however, that the current review is a narrative review, and not a systematic/scoping review. At the end of the review, we discuss limitations with the HTKS measures and necessary future directions such as the need for a systematic review.

## Definitions, conceptualizations, and operationalization

The ubiquity and importance of regulatory skills for conscious activity have contributed to what researchers have called “conceptual clutter” (Morrison and Grammer, 2016). Given the avalanche of terms and frameworks in the literature, we adopt a distinction that conceptualizes EF as primarily reflecting effortful, goal-directed, and largely “top-down” processes under conscious control (Zelazo, 2020). In contrast, self-regulation is broader in scope, encompassing both EF along with more “bottom-up,” emergent influences, including emotional reactivity, motivational states, and automatic physiological responses that support adaptation to environmental demands (Blair and Ku, 2022). EF typically includes multiple cognitive processes including working memory, cognitive flexibility, and inhibitory control (Best and Miller, 2010). As children transition to early learning settings, inhibitory control is especially important because it enables children to follow instructions and engage in learning tasks with minimal distractions. Cognitive flexibility is also essential to preschoolers’ ability to navigate classroom routines, tolerate frustration, and transition between activities (Zelazo and Carlson, 2012). Finally, working memory helps children follow multi-step instructions, complete tasks, and keep several pieces of information active at once (McClelland et al., 2007b). Together, these cognitive processes allow children to focus, pay attention, and remember instructions.

In early childhood, the development of EF occurs gradually and the phenomenon of “unity and diversity” describes how inhibitory control, cognitive flexibility, and working memory are interrelated when children apply their EF to behavior (Miyake et al., 2000), which also aligns with theories such as the Cognitive Complexity and Control framework (Zelazo and Frye, 1998) and the Attentional Network perspective (Rothbart et al., 2003), which conceptualizes EF in early childhood as a unified system. This conceptual understanding is consistent with empirical work indicating that EF in early childhood is best represented as a unitary construct (Brydges et al., 2014; Carlson et al., 2014; Fuhs and Day, 2011; Laureys et al., 2022; Willoughby et al., 2012; Xu et al., 2013).

Repeated engagement of EF can reduce the effort required to carry out certain behaviors over time, as actions become more efficient and less dependent on active executive control. For example, a young child may initially rely heavily on inhibitory control and working memory to remember to raise their hand before speaking during group activities. With consistent practice and environmental support, this behavior may become more routinized, thereby reducing executive demands and allowing EF resources to be allocated toward more complex learning tasks (Floyer-Lea and Matthews, 2004).

In our work, we have described the HTKS as a measure of “behavioral self-regulation [that] integrates aspects of EF,” namely the functions of attentional shifting, inhibitory control, and working memory (McClelland et al., 2014, p. 2). Overall, we take a pragmatic perspective and view EF as a subset of cognitive processes (Blair and Ku, 2022) embedded within the broader self-regulation framework, acknowledging that no single measure fully captures the complexity of regulatory development (McClelland et al., 2015). For example, when a child engages with the HTKS or HTKS-Kids, EF processes are involved from the outset of the assessment experience, beginning with expectations to sit or stand calmly and attend to the examiner and/or tablet. For many typically developing children, these “assessment context” behaviors, such as sustaining attention and remaining relatively still, have become routinized and require minimal active effort. In contrast, the HTKS tasks explicitly require children to engage EF to inhibit responses and to hold and apply changing rules, skills that are still emerging during early childhood. As commands are presented, some children respond by doing exactly what they are told (e.g., touching their head when instructed), rather than applying the “do the opposite” rule (e.g., touching their toes when told to touch their head). In such cases, the child may be demonstrating self-regulation by following the examiner’s requests and remaining engaged with the task context, while simultaneously demonstrating difficulty with the effective use of EF, as reflected in an incorrect response. In this way, performance on the HTKS reflects the dynamic interplay between executive and regulatory processes, highlighting the challenge of disentangling these constructs in early childhood assessment.

For children with moderate to severe disabilities, the assessment context itself can be dysregulating; all their conscious (EF) effort is devoted to staying calm and safe within the assessment space, and they are not able to take in, much less respond to, the EF demands presented with the task rules (Cameron et al., 2025). It is possible that the task is robustly predictive of academic achievement and teachers’ perceptions of children’s behavior because it encompasses a broad range of bottom-up self-regulation behaviors and top-down EF-driven processes that are all required in the early learning environment (e.g., Blair and Raver, 2015; McClelland et al., 2014). Relatedly, children’s responses on the task are aligned to everyday demands of many classrooms: sit or stand in place, attend to an adult while ignoring distractions, remember increasingly complex directions applied to motor/movement choices, and demonstrate flexibility when the directions change. Given the central role of EF in supporting goal-directed learning, it is not surprising that children’s performance on tasks that draw on EF while requiring the fundamental self-regulatory behaviors expected in school contexts, is predictive of later school and life outcomes (McClelland et al., 2015, 2018).

## Measuring self-regulation

The HTKS is situated within a broader set of measures designed to assess EF and self-regulation in early and middle childhood, which vary in methodology, specificity, and theoretical orientation (McCoy, 2019; St Clair-Thompson and Wen, 2021). These include both direct, performance-based assessments and indirect, questionnaire-based measures that capture overlapping behavioral and cognitive aspects of self-regulation.

Indirect assessments typically rely on adult-report questionnaires completed by teachers or caregivers to capture children’s behavior in everyday contexts. Some measures, like the Child Behavior Rating Scale (CBRS; Bronson, 1994; Chan et al., 2021), broadly assess learning-related self-regulation by asking teachers to rate children’s classroom behaviors, including attention, persistence, and following directions. Other measures are designed to assess more specific components of self-regulation. For example, the Behavior Rating Inventory of Executive Function, Second Edition (BRIEF 2; Gioia et al., 2015), captures differentiated dimensions of behavioral, emotional, and cognitive regulation through both global indices and more narrowband subscales. These rating-based approaches are typically conceptualized as reflecting children’s context-dependent self-regulation, providing valuable insights into everyday child functioning across settings. Consistent with this, indirect and direct measures of self-regulation often show low-to-modest correlations with one another, but similar patterns of association with important developmental outcomes, suggesting that they capture related but distinct aspects of self-regulation (McAuley et al., 2010; Toplak et al., 2013). Accordingly, caregiver- and teacher-reported measures are often best understood as complementary to performance-based assessments, each contributing unique information about children’s functioning.

At the same time, indirect measures are not without limitations. Because they rely on adult perceptions, they may be influenced by rater bias and subjectivity (Cameron et al., 2024; Duckworth and Yeager, 2015; Eberhart et al., 2023; Garcia et al., 2019). In addition, these measures can place time and cognitive demands on educators, particularly in classroom settings with competing responsibilities (Chen et al., 2017; Waterman et al., 2012). Thus, while indirect assessments offer important ecological insight, their use alongside performance-based measures may provide a more comprehensive understanding of children’s self-regulation.

Complementary to, yet conceptually distinct from indirect measures, direct measures aim to assess the behavioral manifestations of children’s self-regulation, often under conditions that are controlled or distinct from the naturalistic environment (e.g., home or classroom). Standardized cognitive assessments, primarily intended for clinical contexts, capture elements of EF within broader profiles of children’s cognitive abilities. For example, the Attention and Executive Functions domains of the Developmental Neuropsychological Assessment II (NEPSY-II) include several subtests dedicated to evaluating components of EF, although many are reserved for children beyond age 5 or 7 years (Korkman et al., 2001). Newer child-centered tools now exist, including EF Touch (Willoughby et al., 2012) and the Early Years Toolbox (Howard and Melhuish, 2017) with strong psychometric properties.

As research on self-regulation in early childhood has grown, many laboratory-based tools have been developed for young children (Carlson, 2005). Often, these tasks are specifically designed to assess specific EF processes. For example, the Day/Night Stroop task targets inhibitory control by requiring children to suppress a prepotent response in favor of a subdominant rule-based response (Gerstadt et al., 1994). The Dimensional Change Card Sort task (DCCS) taps cognitive flexibility by asking children to shift between sorting rules (Zelazo, 2006); similarly, classic working memory tasks, such as Backward Digit Span, which require children to recall sequences of numbers in reverse order, have also been widely used (Lipsey et al., 2017).

EF tasks for children have often demonstrated strong psychometric properties (Lipsey et al., 2017). However, many were designed for laboratory contexts and may not capture the demands children face in real-world learning environments (Salthouse et al., 2003). In addition, multiple tasks are often required to obtain a comprehensive picture of a child’s overall self-regulation skills, which can increase administration time and complexity. To address the need for more comprehensive EF assessment, researchers have combined multiple tasks targeting inhibitory control, working memory, and cognitive flexibility into a standardized battery with a digital format. Such approaches can lengthen administration time, and require specialized materials and controlled testing conditions, which can limit scalability and pose challenges for implementation in educational contexts.

Digitized assessments have also been used to streamline assessment with young children through discrete tablet-based tasks that standardize administration and automate scoring, making them easier for non-researchers, including teachers, to use in naturalistic contexts (Cameron et al., 2024; Didrichsen et al., 2025; Denham et al., 2020; Howard and Melhuish, 2017). For example, EF Touch – a multi-task EF battery - was originally developed in paper-and-pencil flipbook form and later computerized for touchscreen administration (Kuhn et al., 2024). Another example is the NIH Toolbox Early Childhood Cognition Battery (NT-ECCB), which includes a Flanker task for inhibitory control and selective attention and a digitized version of the DCCS for cognitive flexibility (Zelazo et al., 2013). The Minnesota Executive Function Scale (MEFS), in contrast, uses a single adaptive sorting task, derived from the DCCS, that aims to engage children’s inhibition, working memory, and cognitive flexibility within a single paradigm with varying task complexity (Carlson and Zelazo, 2014). This reduction in logistical barriers represents an important step toward improved accessibility for strong assessment measures.

As young children increasingly use technology and tablets within classrooms, including early childhood settings (Fletcher et al., 2014; Rideout and Katz, 2016), performance on these measures may be increasingly relevant to classroom learning experiences. However, digitization does not fully address the challenge of ecological validity, because the tasks themselves may be limited in how well they reflect the dynamic, interactive demands of real-world learning environments, particularly given the origin of these measures as laboratory assessments. Moreover, even when administered within authentic contexts, digital measures may not be culturally relevant or appropriate across diverse populations if task demands, interaction styles, and assumed prior experiences do not reflect children’s cultural and educational contexts (Mehdi et al., 2025). Finally, many tasks still require that administration is completed with a trained examiner, and virtually all require a tablet, which can be a potentially significant expense.

## HTKS progression and evolution

The design of the HTKS was informed by research focused on addressing the limitations of laboratory self-regulation tasks with the goal of more closely approximating behavior in real-world settings (McCabe et al., 2000). The HTKS started as the HTT, or Head-Toes Task, which, as a simple two-rule task, showed both psychometric promise (Cameron Ponitz et al., 2008) and predictive validity for American 4-year-olds’ in mathematics, literacy, and vocabulary (McClelland et al., 2007a). The HTT was inspired by the Head-Feet Task, developed as part of a battery of tasks to ensure that self-regulation measurement was ecologically valid for young children from culturally diverse backgrounds who were attending Head Start (McCabe et al., 2004). The HTT and HTKS present engaging and developmentally appropriate response modalities of gross motor actions; the child stands up to do the task and touches their head, toes, shoulders, and knees. This early work highlighted the equity-laden issue of ecological and cultural validity in EF measurement (Miller-Cotto et al., 2022),

By the time they were 5 years old, most children in our research had mastered the one-part HTT, which provided the impetus for the development of the HTKS, a four-rule, two-part task that both predicted kindergarten achievement and correlated with teacher ratings of children’s classroom self-regulation (Cameron Ponitz et al., 2009). Other researchers helped revise the task to include three parts where the four rules switch, which enabled us to measure self-regulation with variation through age 8 years (Lillard and Peterson, 2011). The three-part HTKS demonstrated reliability and validity with a large and diverse sample and laid the foundation for the measure to be more widely accessible (McClelland et al., 2014). Even so, subsequent research documented consistent floor effects for younger children (ages 3–4 years), leading to the development of the HTKS-R; this revised version includes an additional motor-free introductory section designed to reduce task demands by asking children to *say* “head” when the examiner says “toes” and vice versa (Gonzales et al., 2021; McClelland et al., 2021).

After the challenges created by COVID-19, we also had the opportunity to develop a digital version of the HTKS-R, the HTKS-Kids, for use by preschool teachers (Cameron et al., 2024). We are currently testing the HTKS-Kids with a large sample across four U.S. states, including Spanish-speaking children who were given the task in Spanish. Another recent adaptation, the Head-Toes for Neurodiverse Learners (HTNL), highlights the capacity of some children with moderate to severe disabilities (including autism spectrum disorder, Down syndrome, and physical disabilities) to engage in self-regulation assessment, with findings showing that more than half of children can be successfully assessed using a simplified version of the HTKS-R (Cameron et al., 2025).

Table 1 shows the historical development of these various updates to the HTKS. We note that the “tasks” named in column 1 are not distinct assessments, but rather reflect new parts developed for new populations or administration settings. To avoid confusion, we continue to use the term “HTKS” to describe the tasks except when reporting results for specific studies.

**Table 1:** Historical development of the HTKS. Adapted from “Head-Toes for Neurodiverse Learners: Adapting an Existing Behavioral Self-regulation Assessment,” by CE Cameron et al., 2025 CC BY-NC.

Task name	Description and population	Citation(s)
Head-to-Toes (HTT), one-part version	Two rules (head/toes); initial task version for 4-year-old typically developing children	Cameron Ponitz et al. (2008), McClelland et al. (2007a), and McClelland et al. (2007b)
Head-Toes-Knees-Shoulders (HTKS), two-part version	Up to four rules (head/toes; knees/shoulders); new task section added to expand to include 5-year typically developing children	Cameron Ponitz et al. (2009)
Head-Toes-Knees-Shoulders (HTKS), three-part version	Four rules that switch; new task section added to expand to include up to 8-year-old typically developing children	McClelland et al. (2014)
Head-Toes-Knees-Shoulders-Revised (HTKS-R), four-part version	New verbal-only section for two rules (head/toes); adapted to address floor effect for typically-developing 4-year-olds with prior task’s gross motor responses	Gonzales et al. (2021) and McClelland et al. (2021)
HTKS-Kids, digital version of HTKS-R, four-part version	Two- and then four-rule HTKS-R programmed on a tablet and facilitated by early childhood teachers; includes teacher-led, verbal-only section (1 part), and child-led section (3 parts)	Cameron et al. (2024)
Head-Toes for Neurodiverse Learners (HTNL) – two-part version, engagement items	New first section where children first copy the examiner and then follow the command as given, continuing to HTKS-R, with assessor-rated engagement items; adapted to expand task/avoid floor effect for neurodiverse children, specifically those with motor, attention, and cognitive disabilities	Cameron et al. (2025)

## Executive function and self-regulation measurement with the HTKS tasks

Next, we describe how the HTKS and HTKS-R capture the three major EF components and are ecologically valid measures of self-regulation. While the executive demands of the HTKS and HTKS-R are described below by component, the task requires children to coordinate these skills simultaneously rather than use them in isolation, reflecting how EF is typically applied to behavior in real-world learning environments.

The HTKS tasks assess *inhibitory control* by requiring children to perform the opposite of their natural, automatic response. For example, when instructed to “touch your head,” children must instead, touch their toes. By asking children to replace an automatic action with an action that follows a non-intuitive rule, the HTKS tasks offer a real-world measure of inhibitory control that resembles everyday classroom expectations, such as waiting, following multi-step directions, and controlling impulses during group activities (McClelland and Cameron, 2012).

The HTKS tasks capture *cognitive flexibility* because task demands progressively increase in rule complexity, requiring children to modify previously learned responses as rules evolve (Cameron Ponitz et al., 2008). Shifting demands are most pronounced in later sections of the HTKS and HTKS-R, where children are required to abandon previously learned rules (e.g., touching toes) and adopt conflicting responses (e.g., touching knees when prompted to touch their head) (McClelland et al., 2014).

*Working memory* demands are embedded into the structure of all parts of the HTKS assessments. As the task increases in complexity, children must keep several changing rules in mind simultaneously, such as matching four body-part prompts to four different opposite responses or recalling new rule sets added in subsequent sections (McClelland et al., 2014; Schmitt et al., 2017). Successful performance reflects the child’s ability to retain information while executing a behavioral response, illustrating how the task links cognitive processing with coordinated, regulated motor responses in applied settings.

### HTKS measurement properties and psychometrics

The HTKS and HTKS-R have been widely used in early childhood research, with many studies documenting their reliability, validity, and sensitivity to developmental change. Below, we provide an overview of the psychometric properties of the HTKS assessments.

### Reliability of the HTKS and HTKS-R

Reliability refers to the extent to which a measurement tool produces precise, stable, and consistent results within a given population (Creswell and Creswell, 2018). For both the HTKS and HTKS-R, three forms of reliability, including internal consistency, test–retest reliability, and inter-rater reliability, have been examined across a range of cultural contexts.

Extensive evidence supports the homogeneity of the task’s items. Studies using Cronbach’s alpha and McDonald’s omega have reported coefficients typically ranging from 0.85 to 0.96 for the HTKS (Ahmadi et al., 2025; Cumming et al., 2023; Korucu et al., 2025; McClelland et al., 2014; Sezgin and Demiriz, 2015; Spiegel and Lonigan, 2018; Zhang and Rao, 2017). For the HTKS-R, internal consistency has likewise been high, with alpha coefficients ranging from 0.95 to 0.97 for the total score (Gonzales et al., 2021; McClelland et al., 2019; Merculief et al., 2025; Özcan et al., 2023).

The HTKS has shown acceptable to strong test–retest reliability, with correlations ranging from *r* = 0.52 to 0.88, and the HTKS-R has demonstrated similarly good psychometric properties, with estimates around *r* = 0.80 (Ahmadi et al., 2025; Hee et al., 2018; McClelland et al., 2014; Özcan et al., 2023; Perry et al., 2023). Research has consistently documented high inter-rater reliability, with coefficients ranging from 0.85 to 0.98 (Ahmadi et al., 2025; Connor et al., 2010; McClelland et al., 2007a; McClelland et al., 2007b; Pauli-Pott et al., 2019; Cameron Ponitz et al., 2008; Wanless et al., 2011; Zhang and Rao, 2017).

### Construct validity of the HTKS and HTKS-R

Construct validity refers to whether multiple sources of evidence support the construct it is intended to measure (Creswell and Creswell, 2018; Messick, 1994). Taken together, results across cultural contexts support the structural validity of the HTKS and HTKS-R, with consistent evidence that items load appropriately onto their hypothesized underlying distinct constructs, *and* that items load onto a single “EF” construct. Although some studies have identified acceptable fit for multidimensional solutions, the unidimensional structure is most frequently identified as the optimal and most parsimonious model (Ahmadi et al., 2025; da Silva et al., 2025; Gonzales et al., 2021; Hee et al., 2018). A one-factor solution is theoretically aligned with theories that conceptualize EF in early childhood as a unified system (e.g., the Cognitive Complexity and Control framework; Zelazo and Frye, 1998, and the Attentional Network perspective; Rothbart et al., 2003). This means that a single underlying process appears to drive children’s ability to respond to demands that require managing information and impulses while executing a non-intuitive response.

#### Cross-cultural applicability

Originally developed in the United States, the HTKS tasks have been tested in many international contexts and is at least 31 languages. Across these settings, studies generally report psychometric properties and validity evidence comparable to those observed in U.S. samples, supporting the task’s cross-cultural applicability. For example, research conducted in European contexts, including Portugal and Iceland, documents expected associations between HTKS performance and early academic outcomes (Cadima et al., 2015; von Suchodoletz et al., 2013). Studies in East Asian contexts, including China and South Korea, similarly report adequate reliability and theoretically consistent relations with early literacy and mathematics skills (Lan et al., 2011; Son et al., 2013; Wanless et al., 2011). Research in Turkey also supports strong psychometric functioning of the HTKS and HTKS-R (Özcan et al., 2023; Sezgin and Demiriz, 2015).

While factor analytic techniques provide evidence for the internal structure of a construct, measurement invariance extends this work by evaluating whether that structure and the associated item-construct relations function equivalently across groups, supporting valid comparisons of scores (Karalunas et al., 2020). Although measurement invariance as a concept has long been established, empirical evaluations of HTKS invariance have only recently begun to accumulate, coinciding with the measure’s increasing use across culturally diverse populations. Initial cross-cultural work demonstrated that HTKS scores could be meaningfully compared across U.S. and Norwegian samples, with a two-factor structure providing adequate model fit in both contexts (Lenes et al., 2020). Subsequent studies have expanded this evidence base, supporting invariance across national contexts in samples from the U.S. and Iran and supporting a unidimensional representation of the construct in early childhood (Ahmadi et al., 2026), as well as across gender with a Brazilian sample using the HTKS-R (da Silva et al., 2025).

Complementary to the studies that examined the HTKS’s performance as a single-use assessment, Karalunas et al. (2020) investigated the factor structure of several direct self-regulation measures collectively. In their two-factor model, the HTKS loaded onto a broader working memory factor, along with four similar instruments. Using this multi-measure framework, they found evidence of invariance between typically developing children and those diagnosed with ADHD, suggesting that the underlying factor structure, along with the HTKS’s contribution to it, functioned similarly across these two, distinct groups of children. Collectively, these studies illustrate that measurement invariance represents a useful way to test the HTKS across diverse cultural and developmental contexts.

#### Evidence of validity across cognitive and academic domains

In addition to examining the task’s factor structure, several studies have examined associations between children’s HTKS performance and established EF measures, providing convergent evidence of construct validity. This work shows that children who score higher on the HTKS tasks are likely to score higher on measures of inhibitory control, working memory, and cognitive flexibility, and broader EF measures. Findings are consistent after controlling for key covariates such as child age, gender, Head Start status, ELL status, and parent education (Ahmadi et al., 2025; Gonzales et al., 2021; McClelland et al., 2021; Merculief et al., 2025).

Given these demonstrated associations with core executive processes, HTKS performance has been examined extensively in relation to children’s academic achievement. If the HTKS validly captures EF as theorized, performance on the task would be expected to relate to academic outcomes that draw on executive and self-regulatory capacities. From a developmental perspective, several complementary mechanisms help explain these associations. Children’s current level of EF may shape later academic performance by influencing the development of foundational academic skills, which in turn support or constrain subsequent achievement (Valcan et al., 2020). A related mechanism involves automaticity: through repeated practice, motor, cognitive, and academic skills become increasingly automated, freeing cognitive resources for more complex learning (Floyer-Lea and Matthews, 2004; Wulf et al., 2001). Children with weaker EF may struggle to automatize foundational skills and, as a result, have fewer resources available for higher-level academic tasks (McClelland and Cameron, 2019).

A substantial body of research has documented consistent cross-sectional and longitudinal positive associations between HTKS performance and early academic outcomes across literacy, language, and mathematics (Cameron et al., 2012; Cameron Ponitz et al., 2008, 2009; Gestsdottir et al., 2014; McClelland et al., 2014, 2021; Schmitt et al., 2014, 2017). Similar patterns have been observed across diverse national and cultural contexts (Ahmadi et al., 2026; Fuhs et al., 2015; Uka and Von Suchodoletz, 2016; Wanless et al., 2011), and research on the revised HTKS-R has yielded comparable evidence across preschool and kindergarten (Gonzales et al., 2021; McClelland et al., 2021). Comparative work further strengthens this evidence base; among 12 direct self-regulation assessments, Lipsey et al. (2017) identified the HTKS as one of the strongest predictors of academic performance and growth between 4 and 6 years.

Evidence supporting the validity of the HTKS tasks in predicting children’s academic performance has been synthesized in a meta-analysis of HTKS–academic associations (Kenny et al., 2023). Across 69 studies and nearly 20,000 children (including all reported versions of the measure), results indicated consistently significant associations between children’s HTKS performance across outcomes in literacy, language arts and mathematics. Notably, the magnitude of these associations was comparable to (and in some cases stronger than) those reported in prior meta-analyses examining broader self-regulation and related constructs (e.g., Allan et al., 2014; Dent, 2013; Cortés Pascual et al., 2019; Jacob and Parkinson, 2015; Robson et al., 2020; Spiegel et al., 2021). Although associations were stronger for mathematics, effect sizes did not vary significantly by child age or the timing of HTKS administration relative to academic assessment. Socioeconomic status could not be examined as a moderator due to incomplete reporting across studies. However, country was examined as a moderator, with findings suggesting cross-national stability in the associations. This pattern may reflect the performance-based nature of the HTKS, which reduces reliance on subjective judgments.

Building on this empirical foundation, interest has grown in the practical application of the HTKS tasks in educational settings, particularly for early identification and instructional decision-making. For example, Korucu et al. (2025) examined whether performance on the HTKS-R and its earlier version, the HTKS, in the fall of kindergarten could be used to identify children at risk for lower academic achievement by establishing potential cut scores and their associations with both concurrent and longitudinal academic outcomes. Although findings were preliminary, results suggested that specific performance thresholds on both the HTKS and HTKS-R showed promise for distinguishing children at elevated risk, with the strongest classification accuracy observed for concurrent mathematics outcomes. Together, these findings highlight the emerging potential of the HTKS assessments as brief, performance-based screeners that may support early identification and inform instructional planning during a critical developmental transition, while also underscoring the need for continued research to refine and validate cut scores across populations and contexts.

## Future research in the HTKS

Several limitations of the HTKS tasks have been documented in the literature that should be targeted in future research. First, similar to other self-regulation tasks that tap into EF processes, task impurity poses a challenge (Sparrow, 2012). More specifically, the HTKS is not able to separate the different components of EF. However, recent studies (e.g., Lenes et al., 2020) suggest that easier items with only two rules tend to target inhibitory components, while the harder items in later sections target working memory and cognitive flexibility. Second, some studies using the previous version of the task (HTKS) reported relatively high floor effects for younger children and those with lower skills (Cameron Ponitz et al., 2008; Tominey and McClelland, 2011), an issue that has been addressed in the revised version (HTKS-R; Gonzales et al., 2021; McClelland et al., 2021). Third, there are limited studies on the factor structure of the task. Among the few published studies, researchers have taken different approaches to identify its components—some using sum scores of each section in their factor analyses (da Silva et al., 2025; Gonzales et al., 2021; Lenes et al., 2020), while others used item-based analyses (Ahmadi et al., 2025; Hee et al., 2018). Thus, there is a need for more studies on its factor structure and for consistent methods of extracting factors.

Beyond these research limitations, we note practical limitations that include the need for training and, in some cases, access to a tablet for HTKS-Kids administration. Our prior work has found that after a 1-h training, teachers’ scores and researchers’ scores correlate highly when each give the HTKS-R to the same children; however, even 1-h for training may seem burdensome for early childhood programs. Recent and future work aims to enhance inclusivity, adapt for diverse populations, promote practical application, and leverage digital innovations.

### The HTNL as an extension for neurodiverse young children

Most existing self-regulation measures, particularly direct assessments including the HTKS, have been developed and validated primarily with neurotypical populations (Carlson, 2005; Kenny et al., 2023; Willoughby and Blair, 2011). This gap is particularly concerning for children with disabilities, as it may limit their inclusion in research and access to potentially beneficial interventions. As a result, the absence of appropriate assessment tools becomes an equity issue, inadvertently excluding children who may benefit most from self-regulation support. To address this issue, we developed and evaluated the Head-Toes for Neurodiverse Learners (HTNL), a direct self-regulation assessment designed for young children who require notable learning, behavioral, or physical supports. The study included children with moderate to high support needs, including those diagnosed with autism spectrum disorder, intellectual disabilities, and physical disabilities. This work represents the first comprehensive redesign of the HTKS, incorporating expert-informed modifications to both assessment items and administration procedures and evaluating the revised measure using both psychometric and qualitative methods.

The new HTNL items demonstrated strong internal validity and high inter-rater reliability. Children’s overall HTNL composite scores were also positively correlated with teacher ratings of their social–emotional learning and classroom self-regulation. Together, these findings provide preliminary evidence that the HTNL shows promise as a reliable and valid assessment for children with varying support needs and disabilities (Cameron et al., 2025). Importantly, the HTNL is not disability specific. Rather, it incorporates a flexible design intended to reduce barriers for children whose motor, communication, attention, or cognitive skills may differ from those of populations for whom the HTKS has traditionally been administered. As such, further subgroup-specific validation is needed to extend this work. Additional research is also warranted to determine whether specific task modifications are appropriate based on a child’s unique disability profile.

### HTKS across the lifespan: use with adolescents and older adults

While the HTKS was originally developed for children ages 4 to 8 years, recent work has examined its utility beyond childhood. Growing interest has centered on whether HTKS can validly assess self-regulation across later developmental periods, particularly in diverse contexts. Evidence from cross-cultural samples in the Global South, including Vanuatu, Malaysia, Ghana, and Brazil, suggests that the task retains construct validity through age 13, despite reduced variability and ceiling effects (Dutra et al., 2022). Building on this work, Fernandes et al. (2023) examined older children and adolescents (ages 10 to 15 years) in the U.S. and Brazil, and found that an efficiency score (a scaled ratio of completion time to accuracy) reduced ceiling effects. In the U.S. sample, scores were significantly related to children’s working memory, and in the Brazilian sample, scores were associated working memory and cognitive flexibility measures.

Extending this lifespan perspective, recent work has also explored whether HTKS can be adapted for use with older adults as a potential cognitive screening tool. Cerino et al. (2018) administered the HTKS to community-dwelling adults over the age of 60, adapting the measure to allow seated participation and removing stop rules. Completion time for all items on the three-part HTKS measure was used to maximize variability. Results indicated strong convergent and discriminant validity, with faster completion times being associated with stronger cognitive flexibility, inhibitory control, and attention, as measured by the NIH Toolbox. There were no significant effects with working memory in older adults, which could be due to the restriction in range in the HTKS task compared to the working memory measure or because the HTKS completion time score was most related to other measures involving a timed component (Cerino et al., 2018). Together, these findings suggest that HTKS may have promise for assessment in older adulthood although more work is needed.

### HTKS-kids: an app-based assessment for practical use

Current versions of HTKS are reliable and valid but their reliance on one-on-one administration by a trained adult limits scalability beyond research settings. To address this concern, we adapted the HTKS-R into a gamified, tablet-based version, called HTKS-Kids, designed for early childhood classroom use (Cameron et al., 2024). To reduce teacher burden, most of the task is child-led: Children complete items by listening to pre-recorded audio prompts and tapping on the different body parts (e.g., head or toes) of an animated character. For younger and lower-scoring children, an optional teacher-led section is available that mirrors the verbal-only section of the HTKS-R. This section uses pre-recorded audio prompts and a straightforward response-selection format, requiring minimal teacher effort.

Of note, the task demands of the HTKS-Kids (tapping responses on a tablet) differ from the original HTKS, which requires children to tap their own bodies using gross motor responses. Despite this response modality difference (and other differences), preliminary evidence indicates a positive association (*r* = 0.60) between HTKS-Kids scores obtained with teacher facilitation in the classroom and HTKS-R scores obtained by researchers in an individualized setting, suggesting that both tasks assess a shared set of underlying self-regulatory skills (Cameron et al., 2024). The HTKS-Kids is intended for practical use in educational settings while also addressing equity concerns associated with sole reliance on subjective rating scales. Automated scoring and user-friendly feedback are meant to support educators in interpreting children’s performance by identifying strengths and areas for support. In doing so, efficient performance-based measures such as the HTKS-Kids may reduce reliance on subjective ratings, which have been shown to introduce bias and raise equity concerns (Cameron et al., 2024; Eberhart et al., 2023; Garcia et al., 2019).

Prior work has demonstrated that cultural indicators are often confounded with income level, contributing to observed group differences in self-regulation scores (Cameron et al., 2022; Wanless et al., 2011). At the same time, greater variability is frequently observed within groups than between groups, underscoring the importance of direct assessment approaches that capture individual differences. Accurate assessment is critical for ensuring that children receive appropriate supports when needed and that children with unrecognized strengths have access to the same opportunities as their peers. In this context, the HTKS-Kids aims to provide an educator-friendly assessment that translates children’s self-regulation performance into classroom-relevant information.

The HTKS-Kids represents a step toward more accessible and theoretically grounded self-regulation assessment at the kindergarten transition, offering an integrated measure that taps multiple EF processes simultaneously. Given that early childhood programs are typically required to assess a wide range of academic learning domains, a short (5–7 min) self-regulation task can be more practical than other existing app-based tools that use multiple tasks to comprehensively assess each distinct EF process. More research still is needed to understand whether the original format’s gross motor requirements are a key reason the task is such a powerful predictor of children’s school performance.

## Implications for practice: understanding educators’ roles and teacher burden

In educational settings, researchers have examined how educators can support the growth of children’s self-regulation (Cameron et al., 2024; Fuhs et al., 2013; Maich et al., 2019; McClelland and Cameron, 2012). Meanwhile, it is also important to consider how educators view these skills, especially in the context of administering and scoring assessments given their familiarity with and expectations for each child.

For example, the HTKS-Kids can be used to relieve educators of assessment burden and help them understand their children’s self-regulation and school readiness skills (Cameron et al., 2024). A recent study found that preschool educators liked the HTKS-Kids and found that it provided insights on children, particularly higher-than-expected scores for multilingual learners and children with disabilities (Didrichsen et al., 2025). Educators were also surprised that some children scored lower than they expected. Paired with quantitative results showing teacher differences in their own ratings toward Black children compared with non-Black children (Cameron et al., 2024), but no Black/non-Black differences in HTKS-Kids scores, these early findings point to the possibility that directly assessing children’s self-regulation skills can help educators develop a deeper understanding of individual children, especially those from historically marginalized groups.

Direct assessments can also be used to ground instructional decisions in children’s demonstrated skills, and research has shown that classroom organization and teacher-driven behaviors can support the development of EF and self-regulation (Cameron et al., 2005; Day et al., 2015). When educators have accurate information about children’s self-regulation skills, they can more effectively adjust classroom routines and interactions to promote growth. Thus, feedback on children’s HTKS scores can help educators interpret children’s skills and identify organizational practices that can better support all children. For example, a child who scores high on HTKS but has difficulty in the classroom setting can be supported by increased organizational consistency and more engaging learning activities. This highlights how objective, directly assessed EF information can help teachers align their classroom practices with children’s demonstrated strengths and needs.

Translating feedback into effective classroom supports requires educator knowledge of self-regulation, and deepening educators’ understanding may support their own practices. Often, when the topic of self-regulation is broached with educators, it is through the lens of coping strategies to help them through stressful experiences. Moreover, professional development typically involves base-level knowledge and does not include cognitive strategies to support core self-regulation processes such as cognitive flexibility, problem-solving, and working memory (Whitebread et al., 2019). Without insight about the many processes involved in self-regulation, educators may not have effective tools and strategies to support these skills in children. Tasks like the HTKS can be used to broaden knowledge and understanding of the different aspects of self-regulation that are foundational for school success, and how to best support them in children.

## Conclusion

The HTKS tasks are developmentally appropriate measures of self-regulation for young children, complementing other approaches by emphasizing integrated, performance-based assessment. Within this measurement ecosystem, the HTKS tasks are brief, easy-to-administer, performance-based tasks that integrate multiple EF demands into a single behavioral task. Moreover, the HTKS tasks capture the complexity and interdependence of regulatory processes in a manner that aligns with the expectations children encounter in the classroom (Garon et al., 2008). Robust evidence of strong predictive associations between the HTKS and academic and social outcomes underscores their value as research tools and highlights their relevance for informing educational practice (Cameron et al., 2022; Kenny et al., 2023; McClelland et al., 2014; Schmitt et al., 2017).

Across more than a decade of research, the HTKS tasks have provided researchers with reliable and ecologically relevant tools for studying self-regulation and EF, as well as for developing and evaluating intervention effectiveness. Future work is needed to refine how these tasks can become more accessible to practitioners, support the early identification of children who may benefit from targeted supports, inform the design and timing of interventions, and monitor developmental change across diverse educational contexts. As efforts to promote self-regulation and school readiness continue to expand, the HTKS tasks offer a strong foundation for future research aimed at improving measurement, enhancing inclusivity, and strengthening links between assessment and practice.
